# An intranasally administered adenovirus-vectored SARS-CoV-2 vaccine induces robust mucosal secretory IgA

**DOI:** 10.1172/jci.insight.180784

**Published:** 2024-09-24

**Authors:** Baoqing Sun, Qian Wang, Peiyan Zheng, Xuefeng Niu, Ying Feng, Weijie Guan, Si Chen, Jin Li, Tingting Cui, Yijun Deng, Zhangkai J. Cheng, Yongmei Li, Xinke Zhou, Yi Fang, Wei Wang, Zhongfang Wang, Ling Chen, Nanshan Zhong

**Affiliations:** 1 State Key Laboratory of Respiratory Disease, National Clinical Research Center for Respiratory Disease, Guangzhou Institute of Respiratory Health, The First Affiliated Hospital of Guangzhou Medical University, Guangzhou, China.; 2 Guangzhou National Laboratory, Guangzhou, China.; 3 Center for Drug Clinical Study, The Fifth Affiliated Hospital of Guangzhou Medical University, Guangzhou, China.; 4 Clinical Trial Institution Clinical Research Ward, Peking University People’s Hospital, Beijing, China.; 5 Guangzhou Bio-Island Laboratory, Guangzhou, China.

**Keywords:** COVID-19, Vaccines, Adaptive immunity

## Abstract

**BACKGROUND:**

The level of nasal spike-specific secretory IgA (sIgA) is inversely correlated with the risk of SARS-CoV-2 Omicron infection. This study aimed to evaluate the safety and immunogenicity of intranasal vaccination using Ad5-S-Omicron (NB2155), a replication-incompetent human type 5 adenovirus carrying Omicron BA.1 spike.

**METHODS:**

An open-label, single-center, investigator-initiated trial was carried out on 128 health care workers who had never been infected with SARS-CoV-2 and had previously received 2 or 3 injections of inactivated whole-virus vaccines, with the last dose given 3–19 months previously (median 387 days, IQR 333–404 days). Participants received 2 intranasal sprays of NB2155 at 28-day intervals between November 30 and December 30, 2022. Safety was evaluated by solicited adverse events and laboratory tests. The elevation of nasal mucosal spike-specific sIgA and serum neutralizing activities were assessed. All participants were monitored for infection by antigen tests, disease symptoms, and the elevation of nucleocapsid-specific sIgA in the nasal passage.

**RESULTS:**

The vaccine-related solicited adverse events were mild. Nasal spike-specific sIgA against 10 strains had a mean geometric mean fold increase of 4.5 after the first dose, but it increased much higher to 51.5 after the second dose. Serum neutralizing titers also increased modestly to 128.1 (95% CI 74.4–220.4) against authentic BA.1 and 76.9 (95% CI 45.4–130.2) against BA.5 at 14 days after the second dose. Due to the lifting of the zero-COVID policy in China on December 7, 2022, 57.3% of participants were infected with BA.5 between days 15 and 28 after the first dose, whereas no participants reported having any symptomatic infections between day 3 and day 90 after the second dose. The elevation of nasal nucleocapsid-specific sIgA on days 0, 14, 42, and 118 after the first dose was assessed to verify that these 2-dose participants had no asymptomatic infections.

**CONCLUSION:**

A 2-dose intranasal vaccination regimen using NB2155 was safe, was well tolerated, and could dramatically induce broad-spectrum spike-specific sIgA in the nasal passage. Preliminary data suggested that the intranasal vaccination may establish an effective mucosal immune barrier against infection and warranted further clinical studies.

**TRIAL REGISTRATION:**

Chinese Clinical Trial Registry (ChiCTR2300070346).

**FUNDING:**

Natural Science Foundation of China, Guangzhou Laboratory, The First Affiliated Hospital of Guangzhou Medical University.

## Abstract 2

A two-dose intranasal spray of an Ad5-based Omicron vaccine can induce spike-specific sIgA in the nasal mucosa, which may provide an effective mucosal immune barrier against infection and warrants further clinical investigation.

## Introduction

Most licensed COVID-19 vaccines are administered intramuscularly, which can induce systemic immune responses but not mucosal immune responses in the upper airway. Intramuscular vaccination can reduce severe disease and mortality but is less efficient in blocking infection and transmission (1). As of December 31, 2023, over 70% of the world’s population had received various COVID-19 vaccines, and 13.5 billion doses had been administered globally (2). In the United States, although 79% of the population had completed vaccination via intramuscular injections and 34% had received more than 1 booster as of November 9, 2022, at least 94% of people had been infected at least once, and 65% of people had experienced multiple infections (3). Therefore, more effective vaccines need to be developed for better protection for people who are elderly or at risk, and ideally, such vaccines should be user-friendly for frequent vaccination. WHO posted the Global COVID-19 Vaccination Strategy in a Changing World, updated in July 2022, stating the importance of mucosal immunity in reducing SARS-CoV-2 transmission, which may safeguard against the emergence of new variants and their related health and economic consequences (4).

SARS-CoV-2 infection starts in the upper respiratory tract using its spike protein’s receptor binding domain (RBD) to interact with the angiotensin-converting enzyme-2 receptor on the cell surface. Omicron subvariants have become the dominant circulating strains since the end of 2021 and preferentially infect epithelial cells in the nasal passage (5). Increasing reports showed that the level of nasal or salivary spike-specific secretory IgA (sIgA) is inversely correlated with the risk of subsequent Omicron breakthrough infections (6–8). A vaccine that can induce mucosal immunity, especially sIgA, may more effectively block infection than one that induces only systemic immunity. The nasal tract harbors nasal-associated lymphoid tissue, which is part of mucosa-associated lymphoid tissue and plays an essential role in B cell maturation to plasma cells that produce mucosal sIgA (9). sIgA is made locally as dimeric IgA and transported through the polymeric immunoglobulin receptor located on the basolateral side of epithelial cells for secreting to the mucosal surface as sIgA.

Intranasally administered vaccines have the advantage of being easy to use with good acceptance. Replication-incompetent adenoviruses, including human Ad5, human Ad26, and simian serotype Y25 (SAdY25; ChAdOx1), have been employed as vectors for COVID-19 vaccines administered via intramuscular injection (10–14). However, intramuscular injection may not be the best route for adenovirus-vectored vaccines because the natural tissue tropism of adenovirus is not the muscle. Ad5 is a respiratory non-disease-causing adenovirus with high seroprevalence in the human population (15). We previously demonstrated that intranasal administration of a replication-incompetent Ad5 carrying wild-type spike could induce systemic and respiratory mucosal immune responses in animals and conferred sterilizing-like protection in rhesus macaques challenged with SARS-CoV-2 (16). We recently reported that intranasal booster using Ad5-S-Omicron (NB2155) carrying codon-optimized SARS-CoV-2 Omicron BA.1 spike (EPI_ISL_6640919) could induce broad-spectrum mucosal and systemic immune responses against Omicron BA.1 and BA.5 in mice previously injected with inactivated whole-virus vaccine and conferred protection in mice challenged with BA.1.1 (17). Preclinical safety and toxicity assessment demonstrated that this intranasal vaccine had a good safety profile in animal models. In this study, we aimed to evaluate the safety and tolerability of an intranasal spray of NB2155 in health care workers who worked in a hospital and were at high risk of exposure to Omicron subvariants. The induction of mucosal spike-specific sIgA in the nasal passage and serum-neutralizing antibodies were assessed. Because the study was carried out when the zero-COVID policy was lifted, the infection rate of Omicron BA.5 in the population surged rapidly from less than 1% on December 7, 2022, to over 85% by the end of January 2023. Therefore, the infection rate in participants was also monitored to observe possible protection against infection.

## Results

**Figure 1:**
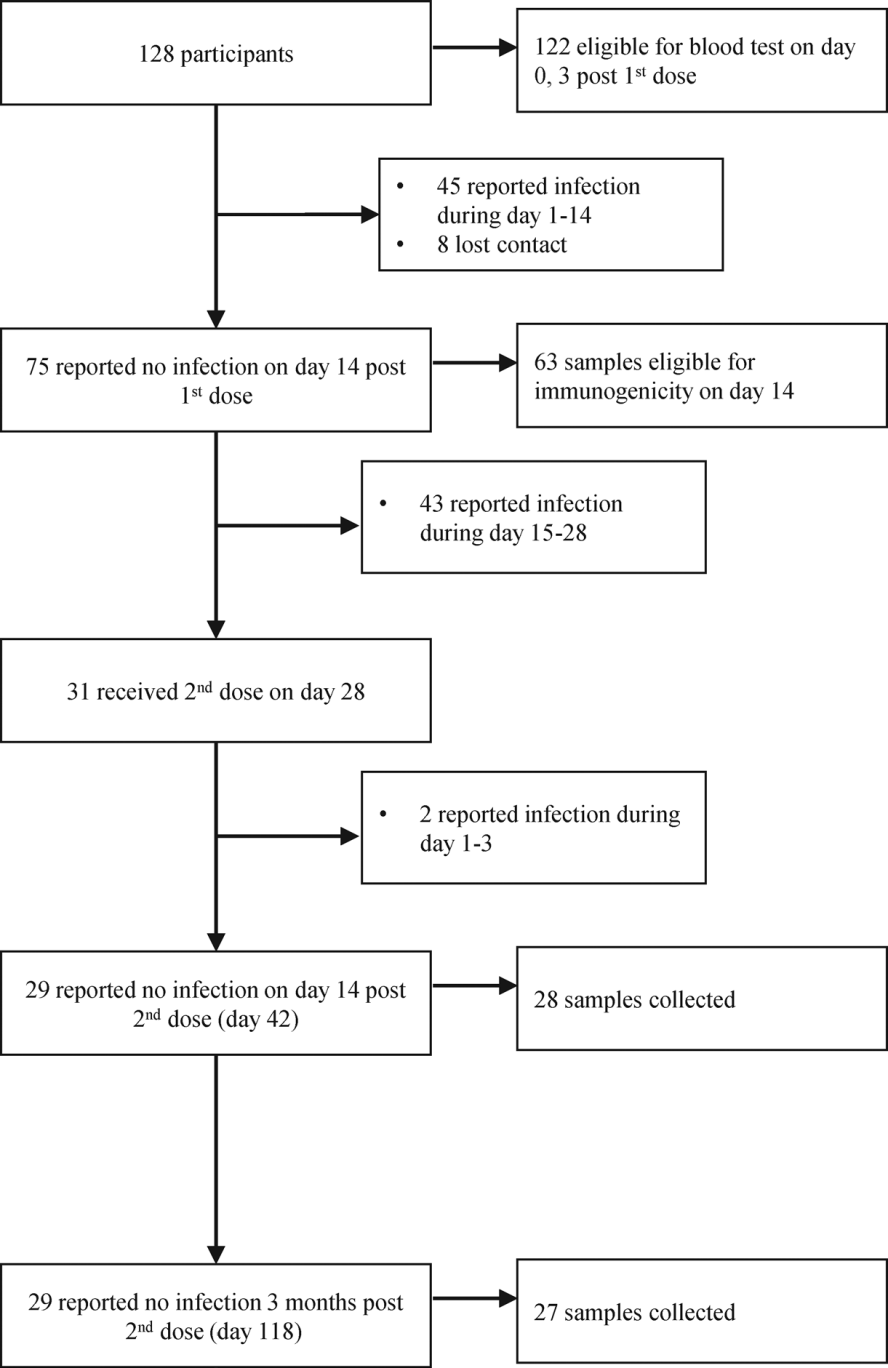
Flow diagram of the study design.

A total of 128 health care workers in The First Affiliated Hospital of Guangzhou Medical University were enrolled to receive the first dose between November 30 and December 6, 2022. A total of 122 participants were eligible for laboratory tests of blood samples on days 0 and 3. On December 7, 2022, the zero-COVID policy was lifted, so the BA.5 infection rate increased rapidly in the population. A total of 45 participants reported infection days 1–14; 63 uninfected participants could provide nasal swabs and blood samples on day 14 after the first dose; 43 participants reported infection days 15–28 after the first dose; 31 participants received the second dose December 28–30, 2022; and 28 and 27 uninfected participants provided nasal swabs and blood samples on day 14 and day 90 after the second dose (Figure 1). All participants were Asian, and the demographics were summarized (Table 1). The median age of the participants was 35 (range 21–55, IQR 30–41), and women accounted for two-thirds.

**Figure 2:**
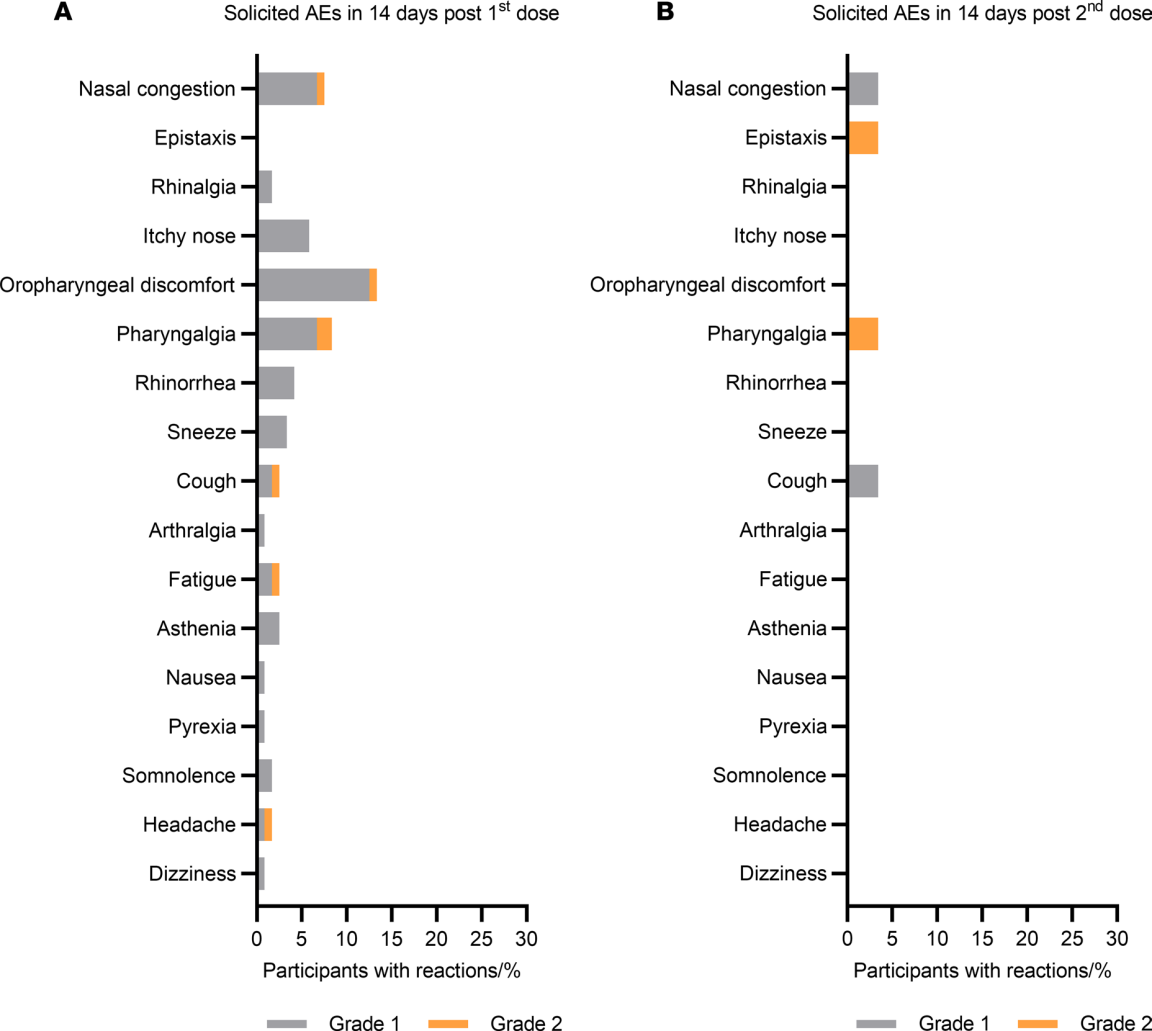
Solicited adverse events following intranasal vaccination. (**A**) The solicited local and systemic adverse events (AEs) were reported by each participant within 14 days after the first dose (*n* = 120). (**B**) The solicited local AEs were reported by each participant within 14 days after the second dose (*n* = 29). No systemic AEs were reported within 14 days after the second dose. Gray shading represents grade 1 (mild) events, and orange shading illustrates grade 2 (moderate) events. The AE categories followed the guidelines promulgated by the Center for Drug Evaluation of the National Medical Products Administration.

No abnormal changes were observed in any laboratory tests — including routine blood; blood chemistry; thyroid function, including thyroid-stimulating hormone, thyroxine, triiodothyronine, and antithyroid autoantibodies; coagulation factor testing including activated partial thromboplastin time, prothrombin time, thrombin time fibrinogen, and D-dimer; and lipase tests — between days 0 and 3. Days 0–14 after the first dose, solicited local and systemic adverse events (AEs) related to vaccination were 25%, mainly grade 1 (23.3%). The most frequently solicited systemic AEs were asthenia (2.5%), fatigue (2.5%), and cough (2.5%). The most common solicited local AEs were oropharyngeal discomfort (12.5%), nasal congestion (7.5%), and pharyngalgia (6.7%). Interestingly, solicited AEs related to vaccination dropped to 10.3% in participants who received the second dose. No systemic AEs were reported after the second dose. The solicited local AEs included nasal congestion (3.4%), pharyngalgia (3.4%), epistaxis (3.4%), and cough (3.4%). All participants reported no severe AEs related to vaccination (Figure 2 and Supplemental Table 1; supplemental material available online with this article; https://doi.org/10.1172/jci.insight.180784DS1).

**Figure 3:**
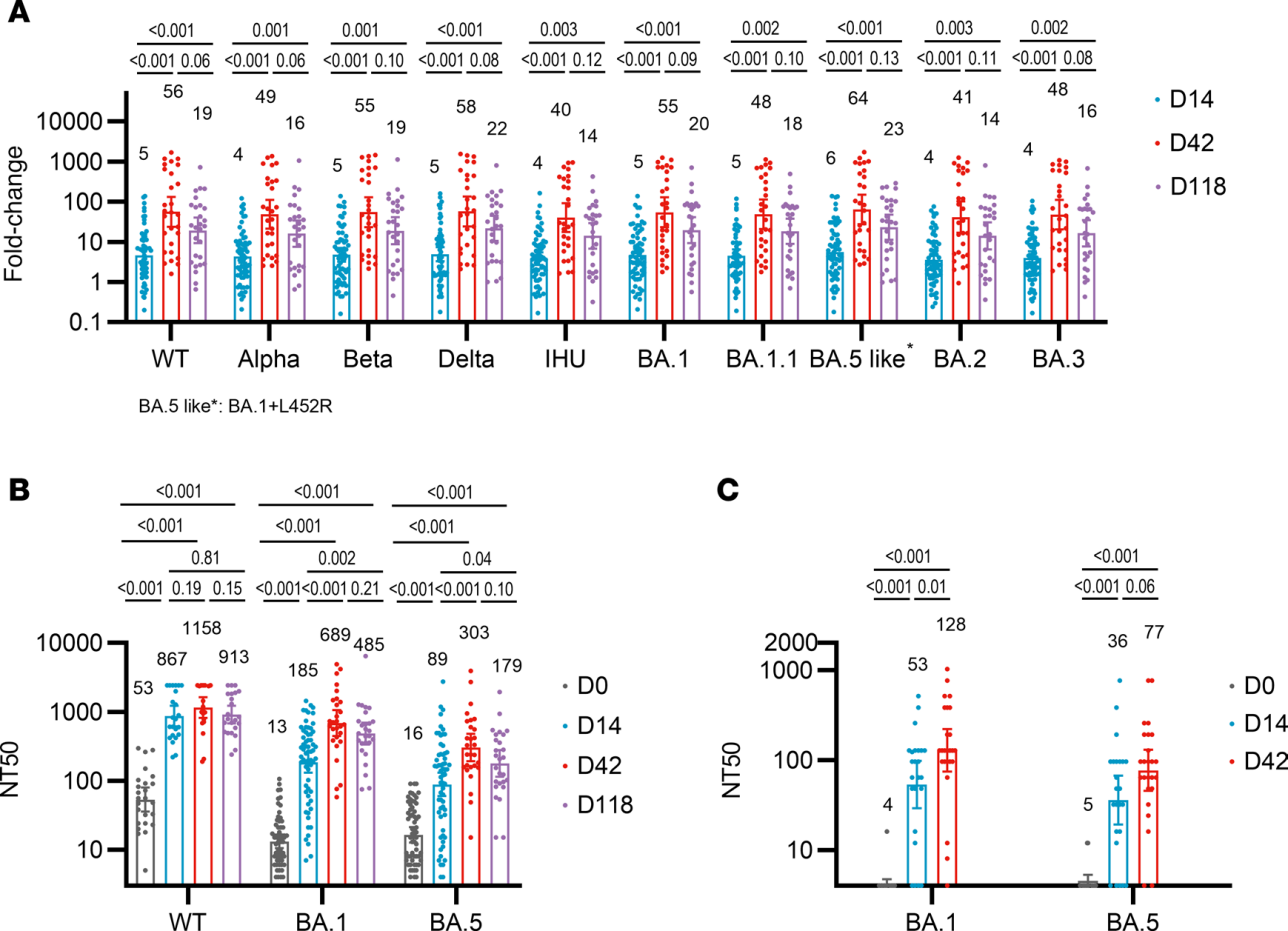
Evaluation of spike-specific sIgA in nasal mucosal lining fluids and neutralizing titer in serum samples. (**A**) The geometric mean fold-increase (GMFI) of spike-specific IgA against spikes of 10 variants in nasal mucosal lavage fluid (NMLF) samples collected from people who reported no infection on days 14 (*n* = 63), 42 (*n* = 28), and 118 (*n* = 27) after the first dose compared with day 0. The second dose was given on day 28 after the first dose. Spike-specific IgA was measured based on the electrochemiluminescence method using V-plex kit (K15585U, Meso Scale Diagnostics; MSD). (**B**) The geometric mean titers (GMTs) against wild-type, Omicron BA.1, and Omicron BA.5 were assessed using a VSV pseudovirus neutralization assay for serum samples collected from people who reported no infection on days 14, 42, and 118 after the first dose (*n* = 63, 28, 27). (**C**) The GMTs against authentic Omicron BA.1 and BA.5 were measured using a cytopathic effect assay for serum samples collected from people who reported no infection on days 0, 14, and 42 after the first dose (*n* = 25, 24, 25). Two-sample *t* tests or Wilcoxon’s tests were used for statistical calculation. *P* values are shown. NT50, neutralizing antibody titer 50, which refers to neutralization titer at 50% inhibition.

The main immunological objective of the study was to evaluate the elevation of mucosal spike-specific sIgA in the nasal passage. Amid the SARS-CoV-2 infection wave, 63, 28, and 27 participants contributed nasal swab samples on days 14, 42, and 118 after the first dose, respectively. We measured the elevation of nasal spike-specific sIgA against spike proteins of 10 SARS-CoV-2 variants, including pre-Omicron variants wild-type, Alpha, Beta, Delta, and IHU and Omicron subvariants BA.1, BA.1.1, BA.5-like (BA.1+L452R), BA.2, and BA.3 (Figure 3A and Supplemental Table 2). On day 14 after the first dose, the average GMFI was 4.5 ± 0.6 (range 3.6–5.6) with 56.5% (range 47.6%–65.1%) conversion rate (more than a 3-fold increase over day 0) against these 10 strains. Intriguingly, on day 14 after the second dose (day 42 after the first dose), the average GMFI dramatically elevated to 51.5 ± 7.5 (range 40.1–64.5) with 89.3% (range 85.7%–92.9%) conversion rates. On day 90 after the second dose (day 118 after the first dose), the average GMFI against 10 strains decreased to 18.2 ± 3.0 (range 14.1– 23.2). Nevertheless, this level was still 4-fold higher than day 14 after the first dose. Therefore, the second dose elicited a much more robust increase of nasal spike-specific sIgA than the first dose.

The serum neutralization titers were measured using vesicular stomatitis virus–based (VSV-based) pseudoviruses (Figure 3B and Supplemental Table 3). The GMTs against wild-type, BA.1, and BA.5 were 53.3, 13.1, and 16.5 before vaccination, which increased to 867.1 (95% CI 612.6–1,227.4), 184.6 (95% CI 131.6–258.9), and 88.5 (95% CI 59.9–130.9) at 14 days after the first dose and further increased to 1,158.1 (95% CI 818.0–1,639.7), 688.4 (95% CI 446.5–1,061.2), and 303.0 (95% CI 192.2–477.7) on day 14 after the second dose. Compared with before intranasal vaccination, the GMFI on day 14 after the first and second doses were 15.6 (95% CI 8.8–27.6) and 21.2 (95% CI 11.0–40.7) against wild-type, 14.3 (95% CI 10.0–20.3) and 47.5 (95% CI 28.4–79.5) against BA.1, and 5.5 (95% CI 3.6–8.2) and 16.8 (95% CI 9.9–28.3) against BA.5, respectively. On day 14 after the second dose, the seroconversion rate (4-fold increase over day 0) was 90.5%, 100%, and 88.9% against wild-type, BA.1, and BA.5, respectively. Three months after the second dose, the GMTs against wild-type, BA.1, and BA.5 were 913.1 (95% CI 675.9–1,233.7), 484.4 (95% CI 335.8–698.8), and 178.9 (95% CI 114.3–280.1). The GMFIs against wild-type, BA.1, and BA.5 were 17.2 (95% CI 9.1–32.2), 34.4 (95% CI 20.9–56.6), and 10.0 (95% CI 5.7–17.6). A cytopathic plaque-forming assay using authentic Omicron BA.1 and BA.5 virus was used to verify the VSV-based pseudovirus neutralization (Figure 3C and Supplemental Table 4). The GMTs against BA.1 and BA.5 were mainly under the detection limit on day 0, increased to 53.2 (95% CI 29.0–97.8) and 35.9 (95% CI 19.2–67.3) on day 14 after the first dose, and further increased to 128.1 (95% CI 74.4–220.4) and 76.9 (95% CI 45.4–130.2) on day 14 after the second dose. On day 14 after the second dose, the seroconversion rate, a 4-fold increase over day 0, was 88.0% against BA.1 and BA.5.

We compared nasal spike-specific sIgA and serum neutralizing antibody responses between wild-type and BA.1. After the first dose, the serum neutralizing titer against wild-type was higher at 1:867 (16.4-fold increase over day 0), whereas the titer against BA.1 was lower at 1:185 (14.2-fold increase over day 0). The serum neutralizing titer against wild-type was significantly higher than against BA.1 (*P* < 0.001), indicating an immune imprinting effect on serum antibodies from previously injected wild-type vaccines. However, such imprinting could be partially overcome by the second dose of BA.1 vaccine. The serum neutralizing titer against wild-type was still higher at 1:1,158, but the magnitude of elevation was only 1.3-fold over day 14 after the first dose (*P* = 0.19), whereas the titer against BA.1 had a more significant elevation to 1:688 with 3.6-fold increase over day 14 after the first dose (*P* < 0.001). In contrast, the magnitude of induction of nasal spike-specific IgA against wild-type and BA.1 was comparable after the first dose (5-fold vs. 5-fold increase over day 0) and the second dose (56-fold vs. 55-fold increase over day 0).

**Figure 4:**
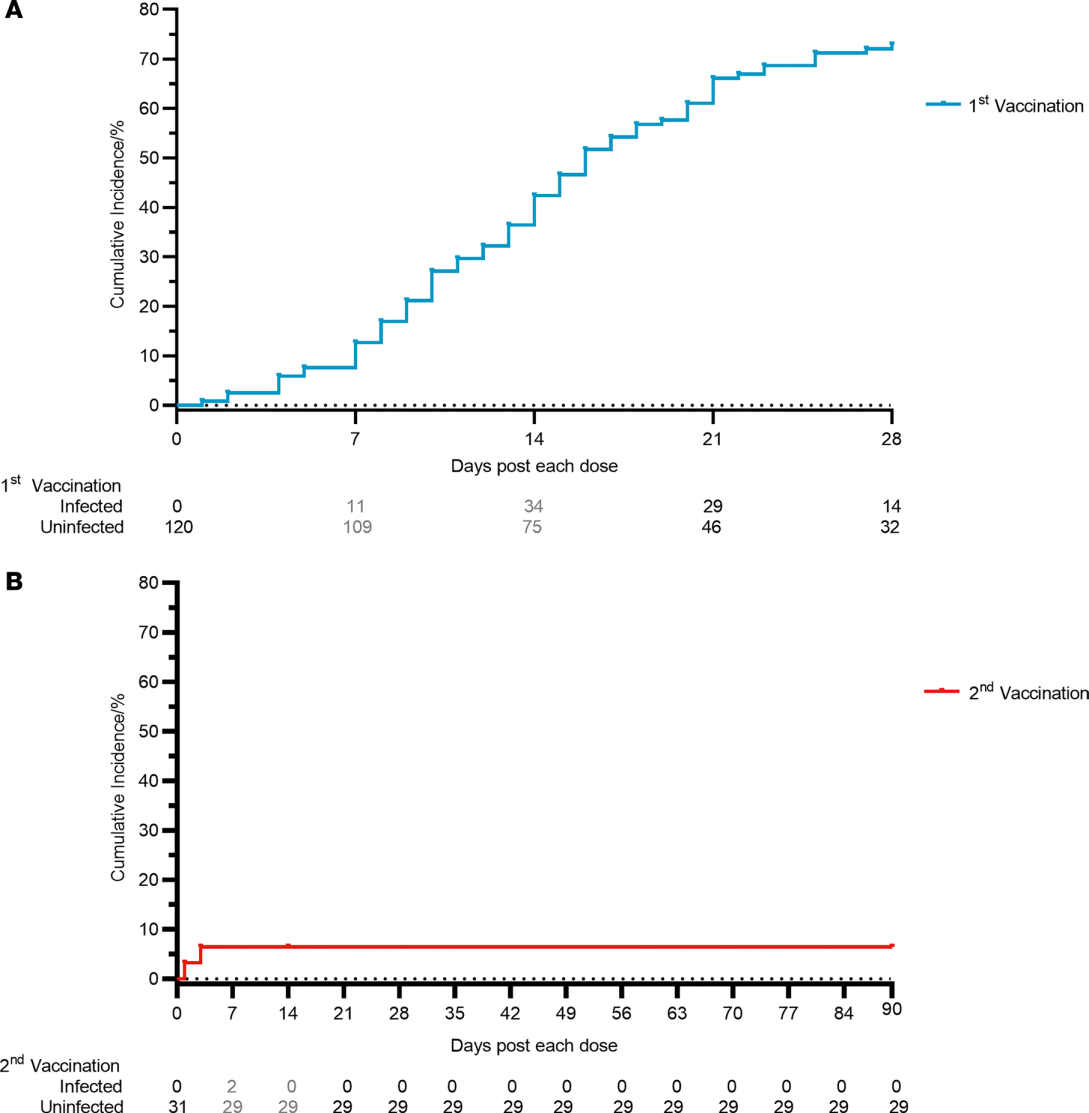
Time-to-infection curve for cumulative infection of SARS-CoV-2 Omicron BA.5 after intranasal vaccination. (**A**) Time-to-infection curve for cumulative infection of SARS-CoV-2 Omicron BA.5 after the first dose. (**B**) Time-to-infection curve for cumulative infection of SARS-CoV-2 Omicron BA.5 after the second dose. The total number of participants and infected participants within a 7-day period was indicated at the end of each week.

The status of SARS-CoV-2 infection was collected and recorded on the day after the first dose to 3 months after the second dose (Figure 4). In people who received 1 dose, 43 out of 75 participants (57.3%) were infected days 15–28 after the first dose (December 14–30, 2022). Overall, 73.3% (88 out of 120) participants were infected between days 1 and 28 after the first dose (November 30–December 30, 2022), suggesting that 1 dose of intranasal vaccination may not have sufficient time and induction of immune barrier to counter infection under a high–viral load environment. Remarkably, among 31 participants who received the second dose (December 28–30, 2022), only 2 participants reported infection on day 1 and day 3 after vaccination. After that, all 29 participants reported no infection for the following 3 months. In the same period, the accumulated BA.5 infection rate in the population of Guangzhou surged from less than 1% on December 7, 2022, to over 85% by the end of January 2023 (18, 19).

It is known that natural infection with SARS-CoV-2 would induce nucleocapsid-specific sIgA in the nasal passage (20–22). To assess possible asymptomatic infections among participants who reported no infection over the study period, we used ELISA to measure nucleocapsid-specific IgA in the NMLFs collected between day 0 and day 118 after the first dose (Supplemental Figure 1A). We first measured the baseline level of nucleocapsid-specific IgA in NMLFs of 111 participants who did not have an infection within 7 days after enrollment and found that the average OD450 value was 0.11 ± 0.08. Therefore, we designated an OD450 value higher than 0.34, i.e., mean baseline value plus 3 times standard deviation, as a cutoff value for having an infection with SARS-CoV-2. Among 63 participants who reported no infection on day 14 after the first dose, no one had an increase of nucleocapsid-specific IgA over the baseline cutoff value. Among 29 participants who provided nasal samples on day 14 after the second dose, 2 participants showed a rise of nucleocapsid-specific IgA over the baseline cutoff value. Among participants who provided nasal samples on day 90 after the second dose, 2 more participants (total 4 out of 29 participants) showed an increase of nucleocapsid-specific IgA over the baseline cutoff value, suggesting that these participants might have an asymptomatic infection. Therefore, most (at least 86.2%) participants who completed 2 doses maintained uninfected status, likely without even asymptomatic infection, for at least 3 months. The immunogenicity results with or without asymptomatically infected participants had no significant difference (*P* > 0.05, Supplemental Tables 5 and 6, and Supplemental Figure 1, B, and C).

## Discussion

This study demonstrated good safety, tolerability, and immunogenicity of Ad5-S-Omicron (NB2155), a replication-incompetent human Ad5 carrying Omicron BA.1 spike, as a monovalent intranasal booster in people who previously received injected inactivated whole-virus vaccines. Most AEs related to this nasal vaccine were mild, with comparable AEs and rates as other intranasal vaccines, such as FluMist, a live-attenuated influenza virus (LAIV), and an influenza virus–vectored COVID-19 vaccine (23, 24). Interestingly, solicited AEs, including oropharyngeal discomfort and nasal congestion, appeared to be much less noticeable after the second dose. Our preliminary observation also suggested that a 2-dose regimen may confer effective protection against Omicron infection even in a high–viral load environment. For participants who reported no infections, we monitored the elevation of nasal nucleocapsid-specific sIgA from day 0 to day 118 after the first dose to identify if there were any possible asymptomatic infections. We found that only 4 out of 29 of those 2-dose participants (13.8%) had a noticeable elevation of nasal nucleocapsid-specific sIgA between day 14 and day 90 after the second dose, suggesting that they might have an asymptomatic infection, though we could not exclude if extremely close contact with the discharged material from infected people may also induce such elevation.

One of the most remarkable findings was that the second dose dramatically induced nasal spike-specific sIgA against 10 SARS-CoV-2 strains ranging from wild-type, Beta, Delta, to all Omicron subvariants tested. Strikingly, the elevation magnitude of 2 doses (51.5-fold increase) was much higher than that of 1 dose (4.5-fold increase), suggesting the importance of the second dose of intranasal booster. Our result differed from clinical studies of 2 nasal vaccines using simian-derived adenoviral vectors, AZD1222 (ChAdOx1 nCoV-19) and BBV154. Using the same MSD method for detecting spike-specific IgA, AZD1222 only generated a >3-fold elevation of nasal IgA against wild-type spike in 18.2% of participants but no IgA against other spikes (25). BBV154 induced a 1.56-fold elevation of salivary IgA against wild-type spike on day 42 over day 0 as measured by ELISA but had no reported effect on nasal IgA (26). Of note, ChAdOx1 is SAdY25 isolated from fecal samples, which may not have the best tissue tropism on nasopharyngeal mucosa (27). BBV154 vaccine used simian Ad36, which was isolated from a nonrespiratory tissue, and the vaccine was delivered by nasal drop rather than intranasal spray (28). We employed human Ad5, initially isolated from the human tonsil, which may have the ideal tissue tropism for the nasopharyngeal tract (15).

Despite the vaccination site being in the nasal passage and our primary goal being to stimulate mucosal immunity, especially spike-specific sIgA against BA.1, we could also detect a modest induction of serum-neutralizing antibodies. The 88.9%–100% conversion rate of serum neutralizing antibodies against wild-type, BA.1, and BA.5 was higher than other intranasal vaccines reported thus far. We found that the elevation and titer of serum neutralizing titers were biased toward wild-type after the first dose of intranasal vaccination, likely due to the immune imprinting effect from the previously injected wild-type vaccines. However, the second dose of the Omicron vaccine could overcome the imprinting effect on serum antibodies. In contrast, nasal mucosal spike-specific sIgA elevation was comparable between wild-type and BA.1, without apparent influence from previously injected wild-type vaccines. These results suggested that the nasal mucosal and systemic immune responses are somewhat compartmentalized. Therefore, assessing nasal mucosal sIgA is a more relevant immunological correlate than serum antibodies for nasal vaccines.

Preexisting immunity against adenovirus is one concern in repeated usage of adenoviruses, especially adenoviruses with high seroprevalence as vaccine vectors. Previous clinical studies using an Ad5-vectored influenza vaccine suggested that intranasal vaccinations were effective in the presence of preexisting anti-Ad5 immunity (29). In our study, the nasal spike-specific sIgA showed a greater elevation (average 10-fold) between the first and second doses. Thus, the second dose remained effective even in the presence of presumably the anti-Ad5 immunity induced by the first dose. We speculate that the presence of limited anti-Ad5 immunity in the nasal passage was insufficient to completely block the influx of a large bolus of Ad5 vaccine. We may thus call each intranasal influx of a large bolus of Ad5 vaccine a “breakthrough vaccination.”

In addition to adenoviruses for COVID-19 vaccines, other viral vectors, such as an LAIV carrying RBD, have been used for intranasal vaccination. In a clinical study (23), 12%–18% positive conversion of RBD-specific IgA in nasopharyngeal swab samples and 10%–22% seroconversion of RBD-specific IgG was observed, but no results on serum neutralization were reported. This vaccine showed 28.2% effectiveness against Omicron infection in people aged 18–59 but has not been tested in children. FluMist, an intranasal seasonal influenza vaccine based on cold-adapted LAIV, is more efficacious than intramuscularly injected vaccines for protecting children (30). LAIV induced a weak serum antibody response but more elevation of mucosal IgA and conferred comparable protection to the intramuscularly injected vaccine in adults (31). So far, we have tested this Ad5-vectored intranasal vaccine only in adults. Future studies should address the immunogenicity and protection effectiveness of different intranasal vaccines in children, adults, and people who are aged. We propose that using different viruses as a vaccine vector; viruses of different serotypes or species of origin; and different antigen selection (RBD or spike), antigen gene design and optimization, expression cassette, formulation, and method of administration (such as intranasal spraying or dropping) may contribute to different immunological outcome and protection efficacy.

The hallmark of our intranasal vaccine was the induction of broad-spectrum spike-specific sIgA in the nasal passage. Although this study did not evaluate the neutralization titers in NMLFs because of the constraint of sample collection, earlier studies by our team and other investigators have shown that nasal IgA in NMLFs possesses neutralizing activities against SARS-CoV-2 (17, 32, 33). It has been established that the risk of breakthrough infection was lower in convalescents with higher levels of nasal or salivary spike-specific sIgA (6, 7). In another study, we purified paired nasal sIgA, serum IgG, and IgA from NMLFs and serum samples and compared their neutralizing activities against both pre-Omicron strains and Omicron subvariants (34). Nasal sIgA can be over 100-fold more potent than serum IgG and IgA in neutralizing Omicron subvariants, including XBB and JN.1.

The primary objective of this study was to evaluate the safety and immunogenicity of intranasal vaccination, which was not designed to assess protection efficacy. The zero-COVID policy was lifted when this study began. Many participants were infected before the vaccination could induce sufficient immune barrier to counter infection, and these infected participants had to drop out of the study. We observed that 43 out of 75 participants (57.3%) were infected days 15–28 after the first dose, December 14–30, 2022, suggesting that 1 dose with 4.5-fold induction of nasal spike-specific sIgA was not sufficient to block infection in an extremely high–viral load environment. Interestingly, 29 participants who received the second dose December 28–30, 2022, whose nasal spike-specific sIgA elevated 51.5-fold, reported no infection in the following 3 months, demonstrating that the second dose is critical for preventing at least symptomatic infection.

In the same period, the accumulated infection rate in the population increased from less than 1% on December 7, 2022, to over 85% on January 18, 2023, according to a public announcement by the Guangzhou Municipal Health Commission (19). A report based on detecting SARS-CoV-2 ORF8 seroconversion also showed that the infection attack ratio was over 80.7% for samples collected January 5–14, 2023 (18). The limitation of this study in assessing protection effectiveness was the lack of a placebo group to monitor the infection rate with the exact timing as the intranasally vaccinated group, which may have an impact given the dynamic epidemic curve between the first and second doses administered. The exclusion of infected participants also lessened the study population. Nevertheless, we believe that the 2-dose regimen is more effective than 1 dose in preventing infection: 1) there was a time overlap between people who received the first dose and the second dose (December 28–30, 2022); 2) except for 2 participants who were infected on days 1 and 3 after the second dose, no more infections were reported as the nasal spike-specific sIgA elevated to 51-fold over the baseline; 3) according to a China CDC report (35), the infection rate based on PCR test started to increase on December 8, 2022; peaked on December 25, 2022, at 29.2%; and then decreased gradually but still was 5.5% on January 23, 2023. When participants received the second dose December 28–30, 2022, the infection rate in the population was at a high level. In particular, these participants were health care workers who worked in a respiratory disease hospital, where many patients with COVID-19 came in for medical care. Therefore, they were constantly exposed to a high–viral load environment. A randomized, placebo-controlled clinical study should be conducted in the future to obtain a more conclusive result.

As the level of nasal spike-specific sIgA decreases over time, the chance of infection may increase. We found that nasal spike-specific sIgA decreased to 18.2-fold over baseline 3 months after the second dose but was still much higher than 4.5-fold after the first dose, keeping these people uninfected. Concurrent with a report that nasal sIgA wanes to the baseline level in 9 months after natural infection (20), our finding that intranasal vaccination–induced spike-specific sIgA descended to 35% of its peak level after 3 months suggests that an intranasal booster may be needed every 6–9 months to maintain effective protection against infection. We tried stratifying the nasal sIgA and serum antibodies between uninfected and infected participants (Supplemental Figure 2). On day 14 after the first dose, the mean GMFI of nasal sIgA for BA.1+L452R (BA.5-like) was 8.0 versus 4.5 between infected and uninfected on days 15–28, whereas the mean GMFI of serum neutralization for BA.5 was 8.3 versus 4.2. Both nasal sIgA and serum neutralization showed a trend of greater induction (nearly 2-fold) among uninfected participants but lack of statistical significance, probably due to relatively small sample size and short observation duration (14–28 days after the first dose) because the second dose was initiated on day 28. On day 14 after the second dose, the mean GMFI of nasal sIgA for BA.1+L452R (BA.5-like) had an 11.5-fold increase from 1:5.6 to 1:64.5. In contrast, serum neutralizing GMFI for BA.5 had only a 3.1-fold increase from 1:5.5 to 1:16.8. Serum neutralizing titer for BA.5 had only a 3.4-fold increase from 1:89 to 1:303, which was lower than intramuscularly injected vaccines that can elicit much higher serum neutralizing titer but still had Omicron breakthrough infections. Intramuscularly injected vaccines can elicit much higher serum neutralizing titer but are limited in preventing Omicron breakthrough infections. Given that accumulated evidence showed that the level of nasal spike-specific sIgA is inversely correlated with the risk of SARS-CoV-2 Omicron infection, we believe that the dramatic induction of nasal spike-specific sIgA after intranasal booster is the key contributor to the defense against infection through the airway.

In conclusion, this study demonstrated that a 2-dose intranasal vaccination regimen using NB2155 can induce spike-specific sIgA in the nasal mucosa and potentially contribute to protection against Omicron infection. Further clinical studies should be conducted to develop a safe, effective, and user-friendly vaccine for preventing infections and blocking virus transmission in the population.

## Methods

Further information can be found in Supplemental Methods.

### Sex as a biological variable.

Sex was not considered a biological variable in this trial as the SARS-CoV-2 immunogenicity acquired by infection or vaccination was not sex biased.

### Study design.

Between November 30, 2022, and December 6, 2022, 128 health care workers, aged 18–59 years old who previously received 2 or 3 doses of intramuscular injection of inactivated whole-virus vaccine 3–19 months ago (median 387 days, IQR 333–404 days), were enrolled. All participants had never been infected with SARS-CoV-2 when enrolling in this study. People with chronic diseases, thrombosis, neurological disorders, seizures, immunosuppression, pregnancy, lactating status, rhinitis or nasal septum defects, nasal cautery, septal defect, and other conditions that could have interfered with the evaluation were excluded. Approximately 28 days after the first dose, participants received the second dose December 27–30, 2022. The study was carried out when the zero-COVID policy was lifted, and the BA.5 infection surged. Therefore, the infections in the participants were also recorded. People who got an infection were excluded from the study. Details of the study are provided in Figure 1. On days 0 and 28, participants received intranasal vaccination with 4 × 10^10^ viral particles/dose/person using a disposable intranasal spray device, TZ-BN30 (Tianzhou Packaging). We administered 0.2 mL of Ad5-S-Omicron (NB2155, provided by Guangzhou nBiomed Ltd.) to each nostril. Participants were assessed for laboratory tests, including routine blood, blood biochemistry, thyroid function, coagulation factor, and lipase tests, on the day before and day 3 after the first dose. AEs were recorded from day 0 to day 14 after each dose. AEs were recorded over the study period. For the immunogenicity analysis, NMLFs and blood samples were collected on days 0, 14, 42, and 118 after the first dose. The NMLFs were collected using a nasal swab (Haishi Hainuo Group, 6323640). The nasal swab was inserted into each nostril and circulated for 10 rounds and then dispensed in saline (0.9% NaCl) containing 0.5% S9 and 0.5% Proclin 300. Serum samples were collected for evaluation of neutralizing activities against SARS-CoV-2 variants. All participants were inquired about SARS-CoV-2 infection or any symptoms throughout the follow-up period.

### Follow-up and data collection.

The primary endpoints for safety and tolerability were assessed by laboratory blood tests, and local and systemic AEs were solicited within 14 days after each dose. All AEs were recorded according to the guidelines for grading for AEs in clinical trials of preventive vaccines issued by the China National Medical Products Administration.

The primary endpoints for immunogenicity were assessed by the elevation of mucosal spike-specific sIgA in NMLFs and serum neutralizing titers. Nasal spike-specific sIgA was detected using a multiplex electrochemiluminescence antibody-binding assay (K15585U, MSD). The serum neutralization titers against wild-type, Omicron BA.1, and Omicron BA.5 were measured using a VSV-based pseudovirus assay. The authentic viruses BA.1 and BA.5 were used to verify the results of the pseudovirus assay.

### Statistics.

The analysis set for safety and tolerability included all participants who received an intranasal spray of NB2155. The frequency and severity of solicited and unsolicited AEs were tabulated in the safety subpopulation, and the denominator for each solicited reaction was the number of participants with nonmissing presence data. Participants who reported SARS-CoV-2 infection before sample collection were excluded from the analysis for immunogenicity and protection evaluation. No imputation was made for missing data. The serum neutralizing GMT and GMFI were calculated. The GMFI was the geometric mean of fold-change or -increase in a set of values relative to a baseline or reference value, as sometimes prevaccination immunogenicity levels are not 0. The 2-sample or paired 2-tailed *t* test or 2-tailed Wilcoxon test was used for statistical calculation using SAS 9.4. *P* ≤ 0.05 was considered significant. Each participant was followed up by visit or telephone and documented. An infection case was defined as having positive results for the SARS-CoV-2 antigen test or nucleic acid test with or without symptoms, and for a few people when no antigen test or nucleic acid test was available, having a history of close contact with infected people and the presence of at least 2 related symptoms such as fever, dry throat, sore throat, cough, and shortness of breath.

### Study approval.

We conducted a single-center, open-label, investigator-initiated trial (IIT) at the National Clinical Research Center for Respiratory Disease, The First Affiliated Hospital of Guangzhou Medical University. The study was conducted following the Declaration of Helsinki and Good Clinical Practice. The study protocol was approved by the Ethics Committee of The First Affiliated Hospital of Guangzhou Medical University (reference number 2022173, 2022179) and was registered with the Chinese Clinical Trial Registry (ChiCTR2300070346, https://www.chictr.org.cn/showprojEN.html?proj=194109). Written informed consent was obtained from all participants.

### Data availability.

The data in this article are provided in the article, supplementary material, or Supporting Data Values file.

## Author contributions

BS was the principal investigator. BS, QW, and PZ designed the trials and the study protocol, and WW helped with the vaccine preparation. YL, Y Fang, and XZ advised as consultants to the protocol. ZW led the laboratory analyses, and PZ, TC, Y Feng, YD, and SC participated in the laboratory tests. XN, WG, JL, and ZJC participated in the site work. QW, PZ, and Y Feng did the statistical analysis. QW drafted the manuscript. NZ and LC reviewed and revised the report. The corresponding authors had full access to all the data in the study and were ultimately responsible for the decision to submit the report for publication.

## Supplementary Material

Supplemental data

ICMJE disclosure forms

Supporting data values

## Figures and Tables

**Table 1:**
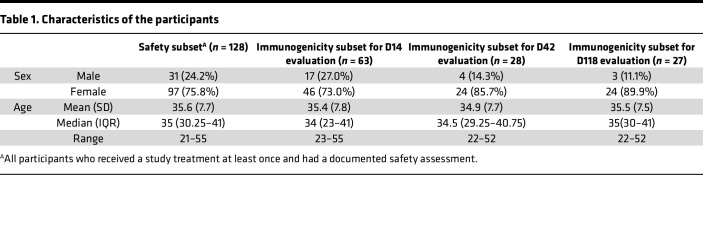
Characteristics of the participants

## Version 1 09/24/2024

Electronic publication
