# Supplementary material for: An intranasally administered adenovirus-vectored SARS-CoV-2 vaccine induces robust mucosal secretory IgA
Source: JCI Insight. 2024 Sep 24;9(18):e180784. doi: 10.1172/jci.insight.180784 (PMC11457852; doi:10.1172/jci.insight.180784)
Supplement: Supplemental data [file jciinsight-9-180784-s006.pdf]

Content

Method.. 2

Sample Collection 2

Electrochemiluminescence binding assay 2

Pseudovirus neutralisation assay 2

Cytopathic plaque-forming assay 3

Anti-Nucleocapsid IgA ELISA assay 3

Supplemental Table 1. Adverse events related to vaccination following each vaccination .. 4

Supplemental Table 2. Geometric mean fold increase (GMFI) and conversion rate of spike-specific sIgA in nasal mucosal lining fluids (NMLFs)... 6

Supplemental Table 3. Serum geometric mean titers (GMT), geometric mean fold increase (GMFI), and conversion rate assessed by pseudovirus neutralization assay......................... 8

Supplemental Table 4. Serum geometric mean titers (GMT), geometric mean fold increase (GMFI) and conversion rate assessed by authentic virus neutralization assay...................... 9

Supplemental Table 5. Geometric mean fold increase (GMFI) and conversion rate of spike-specific sIgA in nasal mucosal lining fluids (NMLFs) after exclusion of participants who had an elevation of nucleocapsid-specific sIgA in NMLFs 10

Supplemental Table 6. Serum geometric mean titers (GMT), geometric mean fold increase (GMFI), and conversion rate assessed by pseudovirus neutralization assay after exclusion of participants who had an elevation of nucleocapsid-specific sIgA in NMLFs 12

Supplemental Figure 1. Evaluation of spike-specific sIgA in nasal mucosal lining fluids (NMLFs) and neutralizing titer in serum samples after exclusion of participants who had an elevation of nucleocapsid-specific sIgA in NMLFs. 13

Supplemental Figure 2. Retrospective evaluation of NMLF or serum antibodies responses in infected and uninfected participants. 14

Method

Sample Collection

For NMLF collection, the participants were use cotton swab(Haishi Hainuo Group (Cat. 6323640) inserted into each nostril and circulated for 10 rounds. The swab was dispensed in saline (0.9% NaCl) containing surfactant 0.5% S9/Tetronic 1307 and preservative 0.5% Proclin 300. Samples were centrifuge at 10000rpm for 5 min and supernatant were transfer to a new sample tube for analysis or storage below -20°C.

For Serum Collection, Whole blood were collected by red topped tubes, and undisturbed at room temperature for 2-4 hours. The samples were then centrifuged at 3000rpm for 15 min. The supernatant serums were transferred immediately to clean ample tube for analysis or storage below -20°C.

Electrochemiluminescence binding assay

SARS-CoV-2 specifically mucosal IgA in NMLF were evaluated by V-PLEX SARS-CoV-2 (K15585U, Meso Scale Discovery, Gaithersburg, MD, USA) according to the manufacturer´s instructions. The assay plates were blocked with 150 µL/well of blocking solution A for 30min at room temperature (RT) with shaking 700-1000 rpm, and washed with wash buffer for 3 times. then the 50µL pre-dilutioned NMLFs with Dilution 100 (R50AA, Meso Scale Discovery, Gaithersburg, MD, USA) were added individually and incubated for 2h at RT with shaking. After 3 times of plates washing with washing buffer, the plates were incubated with 1x SULFO-TAG Anti-Human IgA Antibody (D21ADE, Meso Scale Discovery, Gaithersburg, MD, USA) for 1h at RT with shaking. MSD GOLD Read Buffer (R60AM, Meso Scale Discovery, Gaithersburg, MD, USA) were added after another 3 times of washing with washing buffer, and readed immediately by MSD MESO SECTOR S 600 detection system.

Pseudovirus neutralisation assay

3-fold serial dilutions of serum with a starting dilution of 1:3.3 were co-incubated with 800 TCID50 SARS-CoV-2 pseudovirus supernatants for 1h at 37°C with 5% CO_2. Subsequently, 100 µL of Huh7 cell (JCRB, Cat.#0403) suspension with the density of 3×10^5 cells/ml were then added to the virus-plasma mixture. following a 24h incubation at 37 °C and 5% CO_2, 150µL of supernatant was removed, and 100µL of luciferase detecting regents (Darui Bioscience,Cat.#DR-FLUC-03) was added into each well. Then 150µL of the mixture were transferred to another new microplate after

2 mins incubation to measure the relative light unit(RLU) of luciferase activity by PerkinElmer Ensight. the NT50 were calculated by Reed-Muench method.

Cytopathic plaque-forming assay

The neutralization titer against authentic virus were detected by the cytopathic effect (CPE)-based assay-reduction virus neutralization assay. 2-fold serial dilutions of serum with a starting dilution of 1:4 were co-incubated with 100 TCID50 authentic virus supernatants for 2h at 37°C with 5% CO_2. Subsequently, 1.2×10^4 Vero E6 cells (ATCC, CRL-1586) were added to the virus-plasma mixture. following a 4 days incubation at 37 °C and 5% CO_2, the CPE were examined using a Celigo Imaging Cytometer (Nexcelom Bioscience).

Anti-nucleocapsid IgA ELISA assay

96 well plates were coated with nucleocapsid protein (Sino Biological, 40588-V08B) at a concentration of 0.5 μg/ml in PBS with 100 μl/cell at 4°C over night, and blocked with 1 × PBS containing 0.05% Tween-20 and 5% skim milk for 2 h at 37 °C. NMLFs were prediluted with PBS to 1:8 for detection, and added into the blocked plates for 2h incubation at 37 °C. 100 μl detection antibodies solution were added into each well, and incubated for another 2 hours at 37 °C. The plates were washed with PBST for over 3 times, inverted and tapped on absorbent paper to remove excess liquid each step.

The TMB substrate solution were added into to each well with 100ul/well for 30 minutes at room temperature, and 100 μL of Stop solution were added into each well. The plates were measure at OD 450 as detection wavelength and OD 630 as reference wavelength.

Supplemental Table 1. Adverse events related to vaccination following each vaccination

related AEs	1st vaccination（n=120）									2nd vaccination（n=29）								
	No. of participants	No. of reported cases	Incidence (%)	No. of participants	No. of reported cases	Incidence (%)	No. of participants	No. of reported cases	Incidence (%)	No. of participants	No. of reported cases	Incidence (%)	No. of participants	No. of reported cases	Incidence (%)	No. of participants	No. of reported cases	Incidence (%)
	Grade 1			Grade 2			total			Grade 1			Grade 2			total		
Total	28	66	23.33%	5	7	4.17%	30	73	25.00%	2	2	6.90%	1	2	3.45%	3	4	10.34%
Nasal congestion	8	9	6.67%	1	1	0.83%	9	10	7.50%	1	1	3.45%	0	0	0.00%	1	1	3.45%
Epistaxis	0	0	0.00%	0	0	0.00%	0	0	0.00%	0	0	0.00%	1	1	3.45%	1	1	3.45%
Rhinalgia	2	3	1.67%	0	0	0.00%	2	3	1.67%	0	0	0.00%	0	0	0.00%	0	0	0.00%
Itchy nose	7	7	5.83%	0	0	0.00%	7	7	5.83%	0	0	0.00%	0	0	0.00%	0	0	0.00%
Oropharyngeal discomfort	15	16	12.50%	1	1	0.83%	15	17	12.50%	0	0	0.00%	0	0	0.00%	0	0	0.00%
Pharyngalgia	8	8	6.67%	2	2	1.67%	8	10	6.67%	0	0	0.00%	1	1	3.45%	1	1	3.45%
Rhinorrhea	5	5	4.17%	0	0	0.00%	5	5	4.17%	0	0	0.00%	0	0	0.00%	0	0	0.00%
Sneeze	4	4	3.33%	0	0	0.00%	4	4	3.33%	0	0	0.00%	0	0	0.00%	0	0	0.00%
Cough	2	2	1.67%	1	1	0.83%	3	3	2.50%	1	1	3.45%	0	0	0.00%	1	1	3.45%
Arthralgia	1	1	0.83%	0	0	0.00%	1	1	0.83%	0	0	0.00%	0	0	0.00%	0	0	0.00%

Fatigue	2	2	1.67%	1	1	0.83%	3	3	2.50%	0	0	0.00%	0	0	0.00%	0	0	0.00%
Asthenia	3	3	2.50%	0	0	0.00%	3	3	2.50%	0	0	0.00%	0	0	0.00%	0	0	0.00%
Nausea	1	1	0.83%	0	0	0.00%	1	1	0.83%	0	0	0.00%	0	0	0.00%	0	0	0.00%
Pyrexia	1	1	0.83%	0	0	0.00%	1	1	0.83%	0	0	0.00%	0	0	0.00%	0	0	0.00%
Somnolence	2	2	1.67%	0	0	0.00%	2	2	1.67%	0	0	0.00%	0	0	0.00%	0	0	0.00%
Headache	1	1	0.83%	1	1	0.83%	2	2	1.67%	0	0	0.00%	0	0	0.00%	0	0	0.00%
Dizziness	1	1	0.83%	0	0	0.00%	1	1	0.83%	0	0	0.00%	0	0	0.00%	0	0	0.00%

Supplemental Table 2. Geometric mean fold increase (GMFI) and conversion rate of spike-specific sIgA in nasal mucosal lining fluids (NMLFs)

Variants	Day 14		Day 42 (day 14 post 2nd dose)		D118 (day 90 post 2nd dose)	
	Conversion rate (95%CI)	GMFI (95% CI)	Conversion rate (95%CI)	GMFI (95% CI)	Conversion rate (95%CI)	GMFI (95% CI)
Wildtype	57.1% (44%-69.5%)	4.6(3.2-6.8)	89.3% (71.8%-97.7%)	56.4(23.8-133.4)	77.8% (57.7%-91.4%)	19.2(9.3-40)
Alpha	55.6% (42.5%-68.1%)	4.4(3-6.3)	92.9% (76.5%-99.1%)	48.9(21.4-111.6)	70.4% (49.8%-86.2%)	16.1(7.6-34.3)
Beta	58.7% (45.6%-71%)	4.8(3.2-7.1)	92.9% (76.5%-99.1%)	54.8(23.1-130.2)	74.1% (53.7%-88.9%)	18.9(8.7-40.9)
Delta	57.1% (44%-69.5%)	4.9(3.3-7.4)	85.7% (67.3%-96%)	57.7(24.4-136.6)	85.2% (66.3%-95.8%)	21.8(10.7-44.4)
IHU	57.1% (44%-69.5%)	3.9(2.7-5.5)	89.3% (71.8%-97.7%)	40.1(17.4-92)	70.4% (49.8%-86.2%)	14.1(6.8-29.3)
BA.1	61.9% (48.8%-73.9%)	4.7(3.2-6.8)	89.3% (71.8%-97.7%)	54.5(23-129.1)	74.1% (53.7%-88.9%)	19.6(9.3-41.4)
BA.1.1	54% (40.9%-66.6%)	4.5(3.2-6.5)	89.3% (71.8%-97.7%)	48.4(20.7-113.2)	77.8% (57.7%-91.4%)	18.3(8.9-37.8)
BA.5-like (BA.1+L452R)	65.1% (52%-76.7%)	5.6(3.8-8.3)	92.9% (76.5%-99.1%)	64.5(27.5-150.9)	81.5% (61.9%-93.7%)	23.2(11.3-47.8)

BA.2	47.6% (34.9%-60.6%)	3.6(2.5-5.1)	85.7% (67.3%-96%)	41.4(16.9-101.6)	70.4% (49.8%-86.2%)	14.1(6.5-30.7)
BA.3	50.8% (37.9%-63.6%)	4(2.8-5.8)	85.7% (67.3%-96%)	48.1(20.5-112.8)	77.8% (57.7%-91.4%)	16.4(7.7-34.9)
Mean (SD)	56.5% (5.0%)	4.5(0.6)	89.3% (2.9%)	51.5(7.5)	76.0% (5.0%)	18.2(3.0)
Median (IQR)	57.1% (54.4%-58.3%)	4.6(4.1-4.8)	89.3% (86.6%-92.0%)	51.7(48.2-56.0)	76.0% (71.3%-77.8%)	18.6(16.2-19.5)
Range	47.6%-65.1%	3.6-5.6	85.7%-92.9%	40.1-64.5	70.4%-85.2%	14.1-23.2

Supplemental Table 3. Serum geometric mean titers (GMT), geometric mean fold increase (GMFI), and conversion rate assessed by pseudovirus neutralization assay

Variants	Day 0	Day 14			Day 42 (day 14 post 2nd dose)			D118 (day 90 post 2nd dose)		
	GMT (95% CI)	GMT (95% CI)	GMFI (95% CI)	Positive conversion rate (95%CI)	GMT (95% CI)	GMFI (95% CI)	Positive conversion rate (95%CI)	GMT (95% CI)	GMFI (95% CI)	Positive conversion rate (95%CI)
Wildtype	53.3 (35.6-79.8)	867.1 (612.6-1227.4)	15.6 (8.8-27.6)	87% (66.4%-97.2%)	1158.1 (818-1639.7)	21.2 (11-40.7)	90.5% (69.6%-98.8%)	913.1 (675.9-1233.7)	17.2 (9.1-32.2)	85.7% (63.7%-97%)
BA.1	13.1 (10.5-16.4)	184.6 (131.6-258.9)	14.3 (10-20.3)	77.8% (65.5%-87.3%)	688.4 (446.5-1061.2)	47.5 (28.4-79.5)	100% (87.2%-100%)	484.4 (335.8-698.8)	34.4 (20.9-56.6)	100.0% （86.3%-100%）
BA.5	16.5 (12.8-21.4)	88.5 (59.9-130.9)	5.5 (3.6-8.2)	54% (40.9%-66.6%)	303.0 (192.2-477.7)	16.8 (9.9-28.3)	88.9% (70.8%-97.6%)	178.9 (114.3-280.1)	10.0 (5.7-17.6)	68.0% (46.5%-85.1%)

Supplemental Table 4. Serum geometric mean titers (GMT), geometric mean fold increase (GMFI) and conversion rate assessed by authentic virus neutralization assay

Variants	Day 0	Day 14			Day 42 (day 14 post 2nd dose)		
	GMT (95% CI)	GMT (95% CI)	GMFI (95% CI)	Positive conversion rate (95% CI)	GMT (95% CI)	GMFI (95% CI)	Positive conversion rate (95% CI)
BA.1	<8	53.2 (29.0-97.8)	12.6 (6.8-23.1)	75% (53.3%-90.2%)	128.1 (74.4-220.4)	30.3 (17.4-52.8)	88% (68.8%-97.5%)
BA.5	<8	35.9 (19.2-67.3)	7.8 (4.2-14.4)	66.7% (44.7%-84.4%)	76.9 (45.4-130.2)	16.8 (9.9-28.7)	88% (68.8%-97.5%)

Supplemental Table 5. Geometric mean fold increase (GMFI) and conversion rate of spike-specific sIgA in nasal mucosal lining fluids (NMLFs) after exclusion of participants who had an elevation of nucleocapsid-specific sIgA in NMLFs

Variants	Day 14		Day 42 (day 14 post 2nd dose)		D118 (day 90 post 2nd dose)	
	Conversion rate (95%CI)	GMFI (95% CI)	Conversion rate (95%CI)	GMFI (95% CI)	Conversion rate (95%CI)	GMFI (95% CI)
Wildtype	57.1% (44%-69.5%)	4.6(3.2-6.8)	88.5% (69.8% - 97.6%)	50.1(20.3-123.7)	78.3% (56.3% - 92.5%)	21.4(10-45.7)
Alpha	55.6% (42.5%-68.1%)	4.4(3-6.3)	92.3% (74.9% - 99.1%)	43.2(18.3-102.3)	73.9% (51.6% - 89.8%)	17.5(7.9-38.8)
Beta	58.7% (45.6%-71%)	4.8(3.2-7.1)	92.3% (74.9% - 99.1%)	49.2(19.7-122.9)	78.3% (56.3% - 92.5%)	21.5(9.6-48.2)
Delta	57.1% (44%-69.5%)	4.9(3.3-7.4)	84.6% (65.1% - 95.6%)	51.7(20.7-128.8)	87.0% (66.4% - 97.2%)	24.3(11.4-51.9)
IHU	57.1% (44%-69.5%)	3.9(2.7-5.5)	88.5% (69.8% - 97.6%)	35.1(14.7-84.1)	73.9% (51.6% - 89.8%)	16(7.6-33.7)
BA.1	61.9% (48.8%-73.9%)	4.7(3.2-6.8)	88.5% (69.8% - 97.6%)	45.8(18.8-111.5)	78.3% (56.3% - 92.5%)	21.1(10-44.8)
BA.1.1	54% (40.9%-66.6%)	4.5(3.2-6.5)	88.5% (69.8% - 97.6%)	40.9(17-98.3)	82.6% (61.2% - 95.0%)	19.8(9.6-40.9)

BA.5-like (BA.1+L452R)	65.1% (52%-76.7%)	5.6(3.8-8.3)	92.3% (74.9% - 99.1%)	55.4(22.8-134.7)	87.0% (66.4% - 97.2%)	25.7(12.4-53.3)
BA.2	47.6% (34.9%-60.6%)	3.6(2.5-5.1)	84.6% (65.1% - 95.6%)	35.9(14-92)	69.6% (47.1% - 86.8%)	15.2(6.7-34.4)
BA.3	50.8% (37.9%-63.6%)	4(2.8-5.8)	84.6% (65.1% - 95.6%)	40.7(16.9-98.1)	82.6% (61.2% - 95.0%)	17.6(8.2-37.8)
Mean (SD)	56.5% (5.0%)	4.5(0.6)	88.5% (3.1%)	44.8(6.8)	79.2% (5.7%)	20(3.5)
Median (IQR)	57.1% (54.4%-58.3%)	4.6(4.1-4.8)	88.5% (85.6% - 91.4%)	44.5(40.8-49.9)	78.3% (75.0% - 82.6%)	20.5(17.5-21.5)
Range	47.6%-65.1%	3.6-5.6	84.6% - 92.3%	35.1-55.4	69.6% - 87.0%	15.2-25.7

Supplemental Table 6. Serum geometric mean titers (GMT), geometric mean fold increase (GMFI), and conversion rate assessed by pseudovirus neutralization assay after exclusion of participants who had an elevation of nucleocapsid-specific sIgA in NMLFs

Variants	Day 0	Day 14			Day 42 (day 14 post 2nd dose)			D118 (day 90 post 2nd dose)		
	GMT (95% CI)	GMT (95% CI)	GMFI (95% CI)	Positive conversion rate (95%CI)	GMT (95% CI)	GMFI (95% CI)	Positive conversion rate (95%CI)	GMT (95% CI)	GMFI (95% CI)	Positive conversion rate (95%CI)
Wildtype	53.3 (35.6-79.8)	867.1 (612.6-1227.4)	15.6 (8.8-27.6)	87% (66.4%-97.2%)	1158.1 (818-1639.7)	21.2 (11-40.7)	90.5% (69.6%-98.8%)	951 (687.1-1316.3)	17.1 (8.8-33.2)	85.0% (62.1%-96.8%)
BA.1	13.1 (10.5-16.4)	184.6 (131.6-258.9)	14.3 (10-20.3)	77.8% (65.5%-87.3%)	677 (425-1078.6)	47.9 (27.4-83.8)	100% (86.3%-100%)	463.6 (303.3-708.6)	33.9 (19.2-59.7)	100% (84.6%-100%)
BA.5	16.5 (12.8-21.4)	88.5 (59.9-130.9)	5.5 (3.6-8.2)	54% (40.9%-66.6%)	288.9 (179.2-465.7)	16.3 (9.3-28.6)	88.0% (68.8%-97.5%)	167.3 (100.4-278.7)	9.7 (5.1-18.4)	63.6% (40.7%-82.8%)

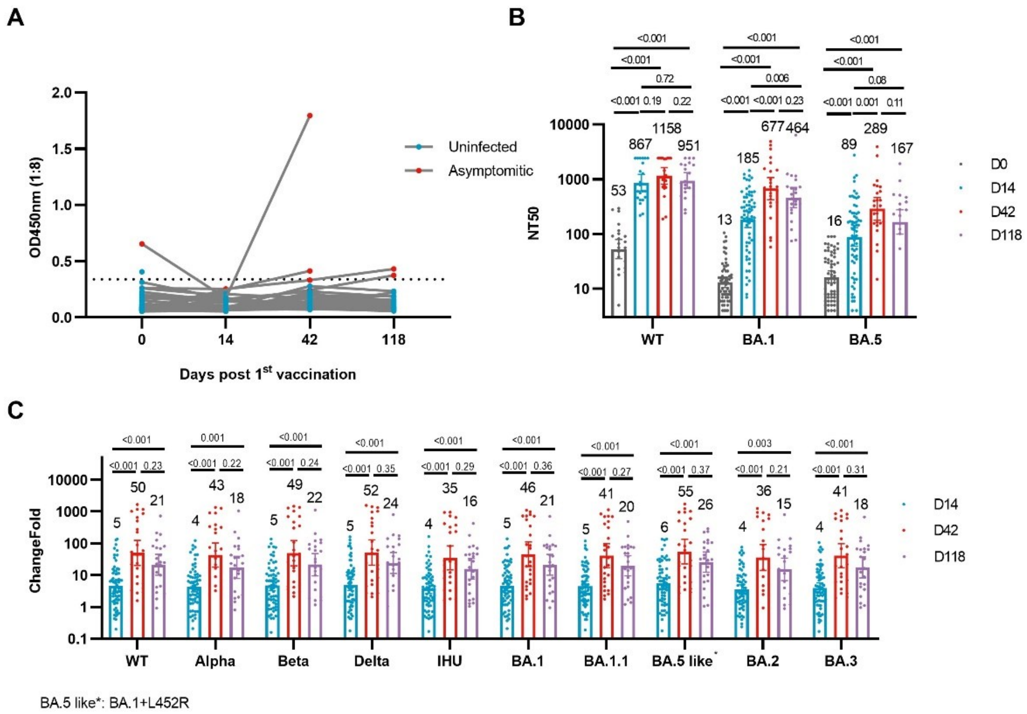

Supplemental Figure 1. Evaluation of spike-specific sIgA in nasal mucosal lining fluids (NMLFs) and neutralizing titer in serum samples after exclusion of participants who had an elevation of nucleocapsid-specific sIgA in NMLFs.

(A) Optical density(OD) against SARS-CoV-2 nucleocapsid IgA antibodies in NMLFs for each self-reported uninfected participants. The dotted line indicated the cutoff value for infection (derived from 111 uninfected subjects). **(B)** The geometric mean titers (GMTs) against WT, Omicron BA.1, and BA.5, were assessed using a VSV-based pseudovirus neutralization assay for serum samples collected on days 14 (n=63.), 42 (n=26), and 118 (n=23) after the first dose. Participants who had an elevation of nucleocapsid-specific sIgA in NMLFs were excluded. **(C)** The geometric mean fold increase (GMFI) of spike-specific sIgA against spikes of 10 variants in NMLF samples collected on days 14, 42, and 118 after the first dose compared to day 0 (n=63, 26, 23). The second dose was given on day 28 after the first dose. Spike-specific IgA was measured based on the electro-chemiluminescent method using MSD V-plex kit (K15585U, Meso Scale). Participants who had an elevation of nucleocapsid-specific sIgA in NMLFs were excluded. Two-sample t tests or Wilcoxon tests were used for statistical calculation. p values are shown.

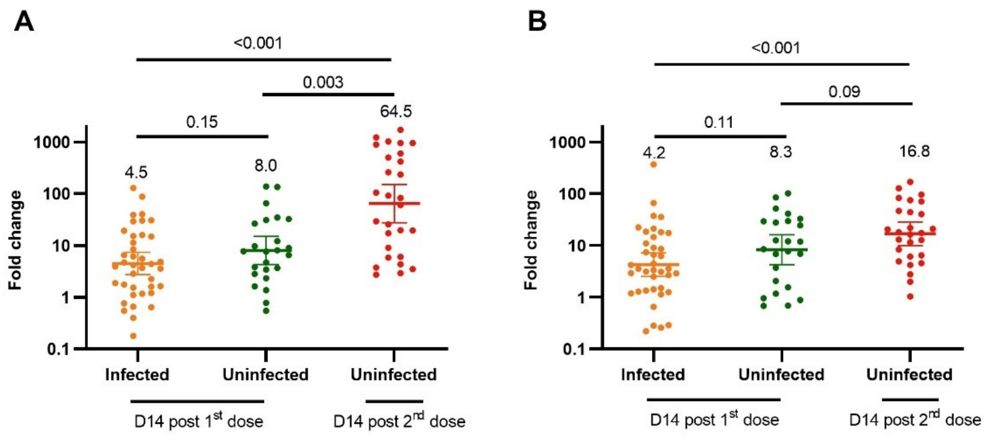

Supplemental Figure 2. Retrospective evaluation of NMLF or serum antibodies responses in infected and uninfected participants.

(A) The geometric mean fold increase (GMFI) of sIgA against BA.5-like (BA.1+L452R) in NMLF samples collected on day 14 after the first and second dose. Shown are participants who were infected (orange plots, n=24) and uninfected (dark green dots, n=39) between days 15-28 after the first dose, and percipients who reported no infection for 3 months after the second dose (red dots, n=28). **(B)** The geometric mean fold increase (GMFI) of neutralizing titer against BA.5 in serum samples collected on day 14 after the first and second dose. Shown are participants who were infected (orange plots, n=24) and uninfected (dark green dots, n=39) between days 15-28 after the first dose, and percipients who reported no infection for 3 months after the second dose (red dots, n=27).
